# Rituximab Use in the Management of Childhood Nephrotic Syndrome

**DOI:** 10.3389/fped.2019.00178

**Published:** 2019-05-10

**Authors:** Mahmoud Kallash, William E. Smoyer, John D. Mahan

**Affiliations:** Division of Nephrology, Nationwide Children's Hospital, The Ohio State University College of Medicine, Columbus, OH, United States

**Keywords:** nephrotic syndrome, rituximab, pediatric, steroid sensitive nephrotic syndrome, steroid resistant nephrotic syndrome

## Abstract

Childhood nephrotic syndrome is a challenging and often persistent renal disorder, and its incidence varies between different ethnicities and regions. Corticosteroids have been the main treatment for decades and are effective in most children with idiopathic NS, although 10–15% of these children become steroid resistant. Furthermore, some initially steroid sensitive children follow a steroid dependent or frequently relapsing course and are therefore at increased risk for developing steroid toxicity. In such children, alternative immunosuppressive medications are used to induce and/or maintain remission of NS. One such drug, rituximab, is a monoclonal antibody directed against the B lymphocyte CD20 marker which induces depletion of B cells, and has shown promising results in the management of NS in children. In this review, we summarize recent studies on the efficacy and safety of rituximab in the different types of childhood nephrotic syndrome, the known and potential mechanisms of action of rituximab, its possible complications and side effects, and the available and potential biomarkers of rituximab activity.

## Introduction

Nephrotic syndrome (NS) is a challenging and often persistent renal disease in children, with an average incidence of 2–16.9 per 100,000 children worldwide, and large variability in incidence between different ethnicities and regions (1–3). For example, NS is six times more common among Asian children than Caucasian children in the United Kingdom, with an overall incidence of 16 new cases per 100,000 children per year (4). In contrast, NS is relatively less common in children of African ethnicity, in whom steroid resistant focal segmental glomerulosclerosis (FSGS) is more common (5).

Corticosteroids are the mainstay for treatment of idiopathic NS (INS). Children with complete resolution of proteinuria with daily prednisone (2 mg/kg/d or 60 mg/M2/d; maximum = 60 mg/d) for 6 weeks are labeled as having steroid sensitive NS (SSNS) and usually have a good clinical outcome. However, at least 50% will develop multiple relapses and may develop steroid dependent NS (SDNS; defined as two consecutive relapses during steroid tapering or within 14 days of cessation of therapy) or frequent relapsing NS (FRNS; defined as at least four relapses per year or at least two relapses within 6 months of initial presentation). These children are often treated with alternative immunosuppressive agents such as cyclophosphamide, mycophenolate mofetil (MMF) and calcineurin inhibitors (CNIs) to improve their clinical course. Response rates to those agents varies among studies, but have been promising in particular for MMF (6) which is generally well-tolerated. In contrast, children who fail to respond to a 4–8 week course of daily corticosteroids are diagnosed with steroid resistant NS (SRNS), at which point CNIs are the main immunosuppressive agents used for treatment (6). For children failing to respond to CNIs, additional alternative immunosuppressive agents are often employed, including MMF, prolonged and/or high-dose intravenous pulse corticosteroids, among other options.

Over the last decade, rituximab has been increasingly used for the management of INS, with quite variable response rates in children with FRNS, SDNS and SRNS. Rituximab is a chimeric anti-CD20 monoclonal antibody that targets CD20 B cells resulting in its significant depletion. It was originally developed to treat individuals with B cell non-Hodgkin's lymphoma, but it also showed significant benefits in children and adults with a variety of renal disorders, including vasculitis, lupus nephritis, and NS. While rituximab is one of the multiple emerging treatments for childhood nephrotic syndrome, this narrative review particularly focuses on the experience to date in the use of rituximab for childhood NS. We will discuss the growing evidence for the efficacy of rituximab in the management of NS, known and possible mechanisms of action, and observed side effects.

## Mechanism of Action of Rituximab in NS

Although the exact mechanism(s) underlying primary NS is not yet well-established, earlier studies suggested that T cells are the main effector cells in the process leading to podocyte foot process effacement and heavy proteinuria (7–10). Thus, the use of a B cell depleting agents may appear to be a puzzling choice. However, more recent evidence suggests that B cells may play a previously unrecognized yet significant role in the development of INS.

### Rituximab Effects on B Cells

B cells, in their primary role as antibody secreting cells, may produce abnormal auto-reactive antibodies that attack podocytes and induce proteinuria, such as antibodies to the soluble urokinase receptor suPAR in FSGS (11, 12), although its role in the pathogenesis of FSGS is not well-established. Another finding that indirectly supports the potential role of B cells in INS is the observation that many NS relapses after successful rituximab therapy occur coincident with recovery of B cells in the plasma (13).

### Effects on T Cells

B cells are also potent antigen presenting cells that provide co-stimulatory signals to T cells, and also produce cytokines that modulate T cell differentiation. Studies in individuals with autoimmune disorders have shown that rituximab causes a significant decrease in the absolute numbers of T cells and T cell subsets, in particular T helper cells (14, 15). Interestingly, some T cells are positive for CD20 receptors, so rituximab may also have direct effects on T cells (16). Studies also have demonstrated that rituximab increases the number of T Reg cells (regulatory T cells) from baseline in individuals with active (nephrotic) membranous nephropathy following rituximab-induced B cell depletion (17), as well as in adults with lupus nephritis (18).

### Cytokine Effects

Another possible mechanism of action for rituximab is cytokine modulation. Abnormal serum cytokine levels have been reported in individuals with NS, including increased IL-13 (19), IL 2, and IL 4 levels (20, 21). In addition, Lai reported significant proteinuria and fusion of podocyte foot processes in IL-13-overexpressing rats (22). Rituximab treatment has also been shown to reduce IL-13 levels in individuals with atopic eczema (23), although the mechanism is not clearly understood; this type of effect has not been explored in rituximab treatment of childhood INS.

### Non-Immunologic Effects

Rituximab may have direct and non-immunological effects on podocytes associated with inducing remission of proteinuria in children and adults with FSGS (24, 25). Researchers have found that rituximab binds to sphingomyelin-phosphodiesterase-acid-like-3b (SMPDL-3b) on the surfaces of podocytes, and suggested that this regulates acid-sphyngomyelinase (ASMase) activity to prevent disruption of the actin cytoskeleton and podocyte apoptosis. In addition, in their work, treatment of individuals with FSGS with rituximab at the time of kidney transplant appeared to prevent recurrent FSGS by modulating podocyte function in an SMPDL-3b–dependent manner.

On the other hand, ofatumumab is a fully humanized antibody against CD20 that has a stronger affinity for CD 20 than rituximab, which theoretically may lead to better clinical responses with less complications. Ravani et al. in 2015 started an open-label, parallel-arm, controlled, phase II, randomized trial comparing ofatumumab to rituximab in children with steroid and CNI dependent nephrotic syndrome (Clinical Trials Registry ID: NCT02394119) (26). Since ofatumumab may not bind to sphingomyelin phosphodiesterase acid-like 3b expressed by podocytes, as has been reported for rituximab, this study may challenge the off-target hypothesis of rituximab directly affecting podocytes. Further studies are needed to confirm the potential of direct effects of rituximab on podocytes.

## Effectiveness of Rituximab in SDNS and FRNS

The first reports of benefits of rituximab in INS were published in the early 2000's and initially consisted of case reports and small case series (ranging from 1 to 24 patients) with SDNS or FRNS. These studies detailed significant responses to rituximab, often with prolonged remission, with many children being able to be weaned off other immunosuppressive drugs (27–31). Despite this, the 2012 KDIGO Glomerulonephritis Guidelines did not strongly endorse the use of rituximab in FRNS or SDNS, since there were no good randomized studies or clinical trials to evaluate its benefits at that time.

Recent published studies now provide stronger evidence to support the benefits of rituximab in FRNS or SDNS (32–41). It can be difficult to directly compare the results of these studies due to their use of different rituximab doses, numbers of doses, and timing of doses delivered. However, these studies reported good clinical responses to rituximab in 80–100% of individuals, with many experiencing significant reductions in their relapse rates for the year following treatment, and many able to be weaned off corticosteroids and/or other immunosuppressive agents.

There has been several well-conducted clinical trials have demonstrated strong and promising results. Ravani et al. conducted a randomized controlled study in 30 children with SDNS who required high dose of steroids to maintain remission (42). These children had non-complicated courses of SDNS without previous exposure to CNIs, and were followed for a minimum of 1 year (median follow up of 22 months). Children in the treatment group (*n* = 15) received one dose of rituximab (375 mg/m2), and although degree of proteinuria was only modestly reduced at 3 months (42% lower than the control group, *P* = 0.58), they had significantly lower doses of prednisone at 3 months (*P* < 0.001) and significantly lower risk of relapses at 6 months, 1, and 2 years after treatment (*P* < 0.01). The median time to relapse after rituximab treatment in this study was 18 months.

Basu et al. conducted an open label randomized clinical trial in India where 120 children with non-complicated courses of SDNS were randomized to receive either tacrolimus for 12 months combined with tapering alternate-day prednisolone or 2–4 weekly infusions of rituximab (375 mg/m2) (43). Frequency of infusions was guided by depletion of B cell count and since all children achieved adequate B cell depletion after the second dose of rituximab, all children received only 2 doses. Rituximab therapy was associated with a higher 12-month relapse-free survival rate compared to tacrolimus (90.0 vs. 63.3%; *P* < 0.001), a longer duration of remission before relapse (40 vs. 29 weeks; *P* < 0.001) and a lower cumulative corticosteroid exposure (27.8 vs. 58 mg/kg 95% confidence interval −77.1 to 43.9). Moreover, the number of infections was twice as high in the tacrolimus group compared to the rituximab group (43 vs. 21%).

In addition, rituximab has also been reported to be have promising results among children with FRNS/ SDNS who had complicated clinical courses (difficult to control NS despite the use of steroid sparing agents) prior to treatment (27, 30, 31, 39, 44–52). Ravani et al. studied 54 children with primary NS dependent on both prednisone and CNI for at least 12 months in an open label randomized controlled trial (53). Children were randomized to 2 groups: Group 1 received rituximab (1–2 doses at 375 mg/m2 per dose) while Group 2 were continued on the standard treatment of prednisone and CNI alone. After 3 months of follow up, proteinuria was 70% lower in Group 1 (95% confidence interval 35% to 86% lower). Rituximab-treated children were also less likely to experience relapses (18.5 vs. 48.1, *P* = 0.029) and more likely to be drug-free at 3 months (62.9 vs. 3.7%, *P* < 0.001).

Another important pivotal trial was conducted in Japan, which contributed to Japanese government approval for the use of rituximab in INS (54). This was a multicenter, double-blind, randomized, placebo-controlled trial where 51 individuals (age 3–38 years) with childhood-onset complicated FRNS or SDNS were randomized into 2 groups: The rituximab group received 375 mg/m^2^ (maximum 500 mg) once weekly for 4 weeks, and the placebo group received placebo infusions at the same frequency. After achieving remission, prednisolone and immunosuppressive drugs were gradually tapered, and subjects were followed up for 1 year. Similar to other studies, this trial showed a lower risk of relapse in the rituximab group (1.54 vs. 4.17 person-years, *P* < 0.0001) and significantly lower total dose of corticosteroids required over the following year (9.12 ± 5.9 vs. 20.8 ± 9.3 mg/m2/day, *P* < 0.0001).

Ahn conducted a multicenter open label trial in Korea where 40 children with CNI-dependent SDNS received a single dose of rituximab at 375 mg/m^2^ (a second dose was administered if the first dose failed to achieve depletion of CD19 cells) and compared the results to 21 controls with SDNS who did not receive rituximab (55). The study showed a remission rate of 74% in the rituximab group compared to 31% in the control group (*P* < 0.003) with a longer duration of remission (9 vs. 2.9 months; *P* = 0 .004) after rituximab. Two other studies that included both pediatric and adult patients with difficult to control SSNS also showed favorable outcomes with the use of rituximab (56, 57).

Taking all those studies together, rituximab appears to be a very effective drug for childhood SDNS or FRNS to maintain remission (average remission duration of 12–20 weeks based on previous studies), reduce the frequency of relapses and enable the reduction or discontinuation of corticosteroids and other immunosuppressive agents. It may also be more cost effective than taking a daily medication with all of its needed monitoring (34, 58, 59) and may contribute to improving the growth of children (56, 60). While other immunosuppressive agents such as MMF and CNIs have proven to be effective in children with FRNS and SDNS, they need to be taken twice daily to maintain remission. Thus, using an IV medication such as rituximab may prove additionally valuable in children where adherence to medications is in question.

## Effectiveness of Rituximab in SRNS

Rituximab is less effective in children with SRNS than in those with SDNS or FRNS, similar to all other alternative treatment agents used for NS. However, in these children, even some improvement of disease control can be of noteworthy clinical significance since many of them have already failed to respond to CNIs and remain floridly nephrotic.

Several years ago, Magnasco conducted a randomized controlled trial in 31 children with SRNS where 16 subjects received CNIs + prednisolone + 2 infusions of rituximab and were compared to 15 subjects who received CNIs + prednisolone alone (61). Unfortunately, none of the children achieved remission (complete or partial) and there was no significant difference in the degree of proteinuria between the rituximab vs. control groups. Perhaps based in part on this poor response, the 2012 KDIGO Guidelines did not recommend rituximab as a treatment option for SRNS, due to both the lack of randomized clinical trials supporting its benefits and the risks for serious adverse events.

However, more studies showing positive responses have been reported since then (62–68). Bagga reported a response rate in 5 of 5 children (100%) with SRNS treated with rituximab (complete remission in 4 and partial remission in 1 patient) (69). In a multicenter study done in Poland, Zachwieja et al. treated 30 children with SRNS who had failed to respond to steroids and CNIs with a single infusion of rituximab (375 mg/m^2^). They found that rituximab improved the response rate compared to CNI only (93 vs. 69%, *P* = 0.09), and enhanced the ability to wean children off both CNIs (77 vs. 50%, *P* = 0.01) and corticosteroids (60 vs. 27%, *P* = 0.005) (70). Kamei et al reported their results of using rituximab (1–4 doses) in ten children who had CNI and methylprednisolone resistant SRNS (71). Rituximab helped induced complete remission in 7 children and a partial remission in another one while 2 children were non—responsive and progressed to end stage renal failure. In a large retrospective study, Sinha et al reported that using rituximab (2–4 doses) in 58 subjects with steroid and CNI resistant nephrotic syndrome (some of them failed also to respond to cyclophosphamide) and 17 of them demonstrated favorable responses (CR in 7 subjects (12%) and PR (17.2%) in another 10) (72). Similarly favorable responses to rituximab have also been reported by other investigators in other studies (55, 73) and in two questionnaire-based studies (74, 75), with complete and or partial remission rates ranging between 29–52%. It is worth noting that the heterogeneity of SRNS itself may explain some of the conflicting response rates. While still unclear, it seems likely that there may well be a subgroup or subgroups of children with SRNS who could be identified early in their clinical course to be far better candidates for rituximab therapy than other subgroups. Such additional studies would be very valuable to enable clinicians to improve the efficacy and reduce the potential toxicity related to rituximab treatment in SRNS.

## Rituximab Use in Relapsing FSGS Post-Transplant:

Primary FSGS recurs in 20–50% of children after kidney transplantation, usually early post-transplant. Although there are no consensus guidelines for the management of FSGS relapses, there have been multiple case reports and small case series about the effectiveness of plasma exchange (76–80). Rituximab has also been used combined with plasma exchange to induce remission in children with FSGS relapses post-transplant, and to help in weaning children off plasmapheresis. The overall responses in these settings have been generally very positive (81–85). Kumar et al treated 8 children with recurrent FSGS post-transplant with 1–4 doses of rituximab (375 mg/m^2^/dose once weekly) after poor responses to plasma exchanges and had good responses in 6 children by 8 months (86). In another study, 2–4 doses of rituximab (375 mg/m2 on weekly) was combined with plasmapheresis and CNIs in 5 children. These investigators found that complete remission was achieved in 2 children (40%), partial remission in another 2 children (40%) and one failed to respond (87). Serious complications were seen in this study, however, including blood stream infections and fatal rituximab-related lung injury.

Overall, rituximab is reasonable to consider in addition to plasmapheresis to treat FSGS relapses post-transplant, although it remains difficult to determine from these studies if the clinical remission of FSGS was induced more by rituximab or plasmapheresis, since those children received combination treatments that included plasmapheresis, steroids, MMF, and high doses of CNIs.

## Factors That May Affect the Clinical Response of Rituximab in NS

As discussed above, rituximab use in children with INS has provided mostly positive results, but there is significant variability in response rates among the different studies. The timing of rituximab administration may play a role in the response rate. Studies have shown that a significant amount of rituximab is lost in the urine when given during a period of heavy proteinuria (estimated to be at least 25% in some cases) (88, 89). For children with FRNS or SDNS, using steroids to induce remission and then giving rituximab when the child is in remission may reduce the amount of medication lost in urine and may help improve the chance of a positive clinical response. There is some experience that supports this concept, with better response rates noted when rituximab was given during remission than during relapse. Several other factors may also affect the response to rituximab, and these are discussed in more detail below.

### Number of Rituximab Doses

The original course of rituximab used for lymphoma patients included 4 doses at 1 week intervals. When it was first used in INS, many pediatric nephrologists used this same approach, although since then many different protocols have been reported, including single-dose, 2-dose, and 4-dose protocols. This variability has hampered the ability to directly compare the efficacy of rituximab among the reported studies. Four weekly doses of rituximab may improve the chances of response by compensating for the amount of medication lost in the urine in some children with NS (90). However, several studies have demonstrated no significant difference in the response rate in children between 1 and 2 doses vs. Three to Four doses (37, 91, 92). Takahashi reported on the efficacy of a single rituximab infusion given routinely, regardless of proteinuria, every 6 months for 2 years in children with SRNS. Although this helped in controlling the NS, it was associated with significant side effects, making further studies important before this approach could be widely recommended (93).

### Dose of Rituximab

In addition to variability in the number of rituximab doses administered, there have also been different rituximab doses used for the management of INS, with most studies have used 375 mg/m^2^ (35, 55, 70, 94). A few studies have been reported with a rituximab dose of 750 mg/m^2^, but this dose has not clearly been associated with a better response rate than the standard dose of 375 mg/m^2^ (37, 95). However, using a lower dose of rituximab (100 mg/m^2^) has been associated with a shorter duration of B cell depletion, and thus appears to increase the risk of earlier relapse (96).

### Concurrent Use of Other Immunosuppressive Agents

Children with SDNS or FRNS, and especially those with SRNS whose disease is difficult to control, are often continued on various doses of other immunosuppressive agents such as MMF or CNIs to try to maintain them in remission and decrease the rate of relapses (97–100). In this context, there is currently a multicenter randomized trial underway to test the benefit of using MMF following rituximab therapy in children with FRNS or SDNS (101).

## Biomarkers of Response to Rituximab

When managing children with NS, the most common biomarkers used to follow treatment responses are the severity of proteinuria and serum albumin level. In some secondary disorders, it may also be useful to follow other specific biomarkers, such as anti-neutrophil cytoplasmic antibodies (ANCA) titers in children with vasculitis or suPAR levels in children with NS relapse post-kidney transplant, to determine response to rituximab treatment (102). Sellam reported that a detectable serum IL-33 level may predict the subsequent clinical response to rituximab in patients with rheumatoid arthritis (103), while Vaknin-Dembinsky reported that specific whole blood microRNA signatures in individuals with neuromyelitis optica might serve as biomarkers for the subsequent rituximab response (104). In addition, the presence of anti-cyclic citrullinated peptide (anti-CCP) antibodies, as well as rheumatoid factor antibodies, are two other plasma biomarkers that have been reported to predict the response to rituximab therapy in individuals with refractory rheumatoid arthritis (105). Checking both serum rituximab levels and anti-rituximab antibody levels are also available, and have been reported in association with rituximab treatment of adults with non-Hodgkin's lymphoma (NHL), chronic lymphocytic leukemia (CLL), and rheumatoid arthritis, although no data are yet available for children with NS.

One of the most commonly followed biomarkers used after rituximab treatment in NS is B cell depletion (typically defined as <1% of lymphocytes), and most studies show very low B cell titers after 1–2 doses of rituximab. Hogan compared 3 groups based on the delivered dose of rituximab: Group 1 received one injection of 100 mg/m2, Group 2 received one injection of 375 mg/m2, and Group 3 received two injections of 375 mg/m2 at Day 0 and Day 7. They found that there was a good correlation between the rituximab dose and the median time to B cell reconstitution (2.5 vs. 5.0 vs. 6.6 months in Groups 1, 2, and 3, respectively). One-year relapse-free survival rates associated with these treatments were also 50, 59, and 72%, respectively (96). These results suggest direct correlations both between dosing regimens and time to B cell reconstitution, and also between time to B cell reconstitution and relapse-free survival.

The results to date of monitoring B cell depletion and reconstitution have been generally encouraging, and have correlated with disease control in rheumatoid arthritis (105, 106), systemic lupus (107), and vasculitis (108, 109). In INS, the evidence is similarly encouraging, and many studies show that most NS relapses occur after B cell recovery (13, 30, 94, 98, 110). The duration of B cell depletion, however, varies significantly among studies, and appears to be influenced by both the rituximab dose and its frequency of administration. Fujinaga reported that early B cell recovery can be associated with a younger age at rituximab treatment, early NS relapse, and stopping other immunosuppressive agents such as MMF (94).

B cell recovery has been used in some protocols to indicate the need for another dose of rituximab to maintain B cell depletion and NS remission (36, 93, 111). Although this approach has been reported to be beneficial in these studies, larger studies are still needed to validate this practice, since it is also known that some individuals can experience NS relapses despite having B cell depletion (36, 49, 112), and repeat dosing of rituximab based on B cell reconstitution rather than NS relapse may expose children to unnecessary rituximab treatment and increase the risk of drug-induced complications. On the other hand, other researchers have reported that some children with NS did not relapse despite documented CD19 recovery, and that the mean time between CD19 recovery and relapse was 4.3 ± 1.0 months (110). In our center, the B cell count is used to help guide the need for further rituximab doses. CD19 levels are checked after the second dose of rituximab, and dose 3–4 is given only if there is no significant depletion in the B cell count.

Another biomarker that can be used to follow the response to rituximab and the risk of relapses is changes in T cell subsets, as shown in the NEPRUTIX study by Boumediene. In this randomized, double blind trial of children with FRNS, NS relapses were associated with significant decreases in both T regulatory cells and IL-2 expression (32). Colucci et al. also found that delayed reconstitution of switched memory B cells was protective against relapse, independent of immunosuppressive treatment (13).

## Side Effects of Rituximab

Rituximab is generally well-tolerated in most published studies of its use in childhood NS. The most common adverse events are mild infusion-related reactions, with a reported frequency between 5 and 53%, which typically resolve with antihistamines and antipyretics as well as slowing the infusion rate (35, 113, 114). Although very few children develop severe anaphylactic reactions or hypotension (30), rituximab can be associated with more serious side effects, and its long-term safety in children with NS is not fully known. Serious complications include arthritis (113), lung injury (113, 115, 116), serum sickness (117), prolonged and severe neutropenia that may develop up to 6 months after infusion (118, 119), inflammatory bowel disease (120, 121) and acute demyelinating neuropathy (122). Expectedly, rituximab also increases the risk of infections, including serious infections such as sepsis (75), Pneumocystis jiroveci pneumonia (123, 124), viral myocarditis (125), fatal hepatitis B infection (126) and severe and/or prolonged hypogammaglobulinemia (127–129). Mortality was recently reported in 5 out of 98 subjects with NS within 1 year of receiving rituximab (~5%) (130).

Another concern regarding rituximab use in children with NS is the lack of long term safety data. The use of multiple courses over multiple years in the same child have been reported by studies at the time of relapses and/ or the time of CD count recovery with no reported significant side effects (31, 36, 42, 52, 57, 100, 131). However, it is not yet known how many doses can be considered safe in children with NS. Repeated doses may also increase the risk of developing anti-rituximab antibodies, and presence of these antibodies has been correlated with more severe infusion reactions (94, 132). Further investigation of the appropriate doses and courses of rituximab is clearly required.

Table 1 lists the side effects of rituximab reported among children with NS, as well as those identified in the broader use of this agent.

**Table 1:** Complications reported with the use of Rituximab.

**Reported complications of rituximab in nephrotic syndrome**
Infections, including: pneumonia, bacteremia, sepsis, osteomyelitis
Mild to moderate infusion reactions, including: rash, tachycardia, nausea, dyspnea, fever
Severe infusion reactions, including anaphylactic reactions or hypotension or death
Viral infection (new vs. reactivation), including: hepatitis B, EBV
Neutropenia
**Reported complications of rituximab in other disorders**
Acute/subacute hypoxemic pneumonia and hypersensitivity pneumonitis (133)
Acute respiratory distress syndrome (133)
Histiocytic necrotizing lymphadenitis or Kikuchi-Fujimoto disease (134)
Myocardial infarctions (135)
Bowel obstruction and perforation (136)

Overall, rituximab is a useful agent in children with NS, but as with any other immunosuppressive agent, its risks should be carefully considered and children should receive close monitoring for infections, especially when rituximab is used with other immunosuppressive agents.

## Possible Future Research Directions Related to Rituximab

We propose 6 future directions for the expansion of knowledge and experience with rituximab in childhood INS:

1. Establishing more effective dosing regimens for rituximab in childhood INS (including the use of concomitant agents) and determining which dosing regimens are most appropriate (i.e., relative efficacy vs. safety) for the different types of NS.
2. Determining rituximab pharmacokinetics and pharmacodynamics in children with NS during both remission and relapse, since such studies have never been adequately performed for rituximab in children.
3. Evaluating the use of rituximab as a second line agent immediately upon diagnosis with SRNS. This approach could reduce the risk of side effects from CNIs, reduce the need for frequent CNI trough levels, and ensure compliance among children with concerns for poor adherence.
4. Exploring the potential role for rituximab as a first line agent in INS. Rituximab could be considered as a potential first line treatment for NS in children with high risk for steroid toxicity, such as children and adolescents with obesity or metabolic bone disease.
5. Identifying and validating biomarkers that predict the clinical response and/or drug toxicity to rituximab in children with NS.
6. Establishing biomarkers that predict a long-lasting response to rituximab in childhood NS (such as serum cytokine levels), as well as the optimal timing for re-dosing to maintain NS remission (e.g., at time of relapses vs. time of recovery of serum biomarkers) and minimize cumulative steroid exposure.

## Conclusions

Rituximab is an effective and increasingly used treatment option to induce or prolong clinical remission in children with NS. Rituximab may very well-exert its beneficial effects through multiple mechanisms of action. There is now growing evidence to support its efficacy and safety in children with SDNS or FRNS in inducing prolonged remission, enabling reduced corticosteroid exposure and side effects. In children with SRNS, there is evidence of beneficial effects in a smaller but sizable percentage of children. Rituximab is generally well-tolerated in these children, but it requires close monitoring for possible side effects which can be serious or fatal in some cases. With continued efforts to standardize rituximab treatment regimens within the pediatric nephrology community, combined with systematic prospective data collection regarding treatment doses and courses, outcomes, and complications, the use of rituximab can almost certainly be made both more efficacious and safer for children with NS in the future.

## Author Contributions

MK helped conceive the project and scope, completed literature review, composed first draft, and helped make final edits to produce the final manuscript for submission. WS contributed to design the project, refined multiple drafts, finalized the future research section, and helped make final edits to produce the final manuscript for submission. JM helped conceive the project and scope, refined multiple drafts, generated the first draft of the future research section and helped make final edits to produce the final manuscript for submission.

### Conflict of Interest Statement

The authors declare that the research was conducted in the absence of any commercial or financial relationships that could be construed as a potential conflict of interest.

## Abbreviations

NS: Nephrotic syndrome
INS: Idiopathic NS: INS
CNIs: Calcineurin inhibitors
MMF: Mycophenolate mofetil
SSNS: Steroid sensitive nephrotic syndrome
SDNS: Steroid dependent nephrotic syndrome
FRNS: Frequent relapsing nephrotic syndrome
SRNS: Steroid resistant nephrotic syndrome
FSGS: Focal segmental glomerulosclerosis
suPAR: Soluble urokinase-type plasminogen activator receptor.
